# bHLH003, bHLH013 and bHLH017 Are New Targets of JAZ Repressors Negatively Regulating JA Responses

**DOI:** 10.1371/journal.pone.0086182

**Published:** 2014-01-23

**Authors:** Sandra Fonseca, Patricia Fernández-Calvo, Guillermo M. Fernández, Monica Díez-Díaz, Selena Gimenez-Ibanez, Irene López-Vidriero, Marta Godoy, Gemma Fernández-Barbero, Jelle Van Leene, Geert De Jaeger, José Manuel Franco-Zorrilla, Roberto Solano

**Affiliations:** 1 Departamento de Genetica Molecular de Plantas, Centro Nacional de Biotecnología–Consejo Superior de Investigaciones Científicas, Campus Universidad Autónoma, Madrid, Spain; 2 Genomics Unit, Centro Nacional de Biotecnología–Consejo Superior de Investigaciones Científicas, Campus Universidad Autónoma, Madrid, Spain; 3 Department of Plant Systems Biology, VIB, Gent, Belgium; 4 Department of Plant Biotechnology and Bioinformatics, Ghent University, Gent, Belgium; Institut Jacques Monod, France

## Abstract

Cell reprogramming in response to jasmonates requires a tight control of transcription that is achieved by the activity of JA-related transcription factors (TFs). Among them, MYC2, MYC3 and MYC4 have been described as activators of JA responses. Here we characterized the function of bHLH003, bHLH013 and bHLH017 that conform a phylogenetic clade closely related to MYC2, MYC3 and MYC4. We found that these bHLHs form homo- and heterodimers and also interact with JAZ repressors *in vitro* and *in vivo*. Phenotypic analysis of JA-regulated processes, including root and rosette growth, anthocyanin accumulation, chlorophyll loss and resistance to *Pseudomonas syringae*, on mutants and overexpression lines, suggested that these bHLHs are repressors of JA responses. bHLH003, bHLH013 and bHLH017 are mainly nuclear proteins and bind DNA with similar specificity to that of MYC2, MYC3 and MYC4, but lack a conserved activation domain, suggesting that repression is achieved by competition for the same cis-regulatory elements. Moreover, expression of *bHLH017* is induced by JA and depends on MYC2, suggesting a negative feed-back regulation of the activity of positive JA-related TFs. Our results suggest that the competition between positive and negative TFs determines the output of JA-dependent transcriptional activation.

## Introduction

Jasmonates (JAs) are fatty acid derived molecules, ubiquitous in the plant kingdom and structurally similar to animal prostaglandins. They regulate many plant cellular and developmental processes such as cell cycle, plant growth, fertility, root elongation, gamete development, trichome initiation, and senescence [1]–[7]. JAs are also potent alert signals that trigger the activation of responses to different stresses, such as pathogens, herbivores, mechanical wounding, or exposure to ozone or drought [8], [9]. Such a variety of responses require a tight regulation at different levels including biosynthesis, hormone accumulation, perception and signal transduction.

In Arabidopsis, the bioactive hormone, (+)-7-iso-jasmonoyl-L-isoleucine (JA-Ile), is perceived by a receptor complex that comprises CORONATINE INSENSITIVE1 (COI1, an F-box component of SCF-type E3 ubiquitin ligases) and a member of the JASMONATE ZIM DOMAIN (JAZ) protein family [10]–[17]. In addition to co-receptors, JAZ are repressors of the TFs regulating JA-responses and recruit the general co-repressors TOPLESS (TPL) and TPR (TOPLESS Related Proteins) either directly or through the adaptor protein NINJA [10], [15], [17]–[23].

Upon hormone recognition, JAZ are ubiquitinated and degraded by the proteasome [10], [15]. TFs are then released and activate transcription. The bHLH TF MYC2 plays a central role in JA signaling and was the first TF identified regulating a subset of JA-responsive genes [24]–[26]. *jin1-2/myc2* mutant was only partially impaired in JA responses, which suggested that other TFs should act additively or redundantly to it [26]. In fact, its closest protein homologs MYC3 and MYC4 were found to share redundant functions with MYC2 in the regulation of JA-regulated gene expression, root growth and pathogen and insect resistance [27]–[29]. Remarkably, the triple mutant *myc2myc3myc4* is completely depleted of glucosinolates and therefore, fully susceptible to insects [28]. MYC2, MYC3 and MYC4 act cooperatively with MYB TFs to activate glucosinolate biosynthesis in response to JA adding another level of complexity to the regulation of JA responses [30].

Additional targets of JAZ from distinct TF families have been described in Arabidopsis. This is the case of the bHLH, Glabra3 (GL3), Enhancer of Glabra3 (EGL3) and Transparent Testa8 (TT8), involved in trichome formation and anthocyanin accumulation, and Inducer of CBF Expression1 (ICE1) and ICE2 involved in cold signaling [31]–[33]; the R2R3 MYBs, Glabra 1 (GL1) and PRODUCTION OF ANTHOCYANIN PIGMENT1 (PAP1) and MYB21 that are also involved in trichome development, anthocyanin biosynthesis and male fertility [33], [34]; and the ethylene related TFs, EIN3 and EIL1 which are involved in ET signaling and defense against necrotrophs, where the JA/ET cross-talk plays a relevant regulatory role [26], [35], [36].

Several mechanisms contributing to repress the JA pathway have been recently reported. Most JAZ genes are transcriptionally induced by JA, thus providing a negative feed-back mechanism for repression. Moreover, alternative splicing of some JAZ genes give rise to truncated forms of JAZ without the Jas domain. This domain is responsible for the interaction with COI1 and, therefore, such truncated forms are resistant to degradation but still repress the TFs [17], [37]–[39]. Similarly, JAZ8 lacks the canonical Jas degron and is unable to interact with COI1, being therefore a stable protein that behaves as a constitutive repressor [23]. The catabolism of the bioactive hormone also contributes to shut-down the JA signal. The fatty acid ω-hydroxylase CYP94B3 is induced in response to JA and converts the bioactive JA-Ile in the inactive 12-hydroxy-JA-Ile (12OH-JA-Ile) [40]–[42]. The activation of parallel metabolic pathways that converts JA in inactive molecules as JA-glucose esters [43], 12-hydroxy-JA (12-OH-JA) and its sulfated and glycosylated derivatives [44], [45], volatile methyl-JA (MeJA), and JA-amino acid conjugates other than JA-Ile [46] might play a complementary role on the process of active hormone depletion.

The existence of all these mechanisms of repression underscores the importance of a tight and timely regulation to prevent a harmful activation of the JA-pathway. Here we describe another repression mechanism based on competition of positive and negative TFs for their cis-regulatory elements in the promoters of JA-regulated genes. We found that a group of bHLH TFs (bHLH003, bHLH013 and bHLH017), phylogenetically close to MYC2, MYC3 and MYC4, homo- and heterodimerize and interact with JAZ proteins *in vivo*. In contrast to MYCs, the three bHLHs described here behave as transcriptional repressors of all JA-related phenotypes tested. Our results are in line with very recent works on these TFs [47]–[49], showing that they bind efficiently to the G-box and that competition for DNA-binding might underlay the molecular mechanism of repression.

## Results

### bHLH003, bHLH013 and bHLH017 are new targets of JAZ repressors

We previously described the domain in MYC2 responsible for the interaction with JAZ repressors (the JID domain [28]). BLAST searches revealed that this domain was conserved among several bHLH proteins including MYC3, MYC4, GL3, EGL3, TT8, bHLH028, bHLH003, bHLH013 and bHLH017 [28]. Interaction between JAZ proteins and MYC3, MYC4, GL3, EGL3 and TT8 has been recently characterized, whereas bHLH028 does not interact with JAZs [27]–[29], [33]. Thus, we focused on the characterization of bHLH003, bHLH013 and bHLH017, which conform a phylogenetic clade closely related to MYC2, MYC3 and MYC4 [28].

**Figure 1:**
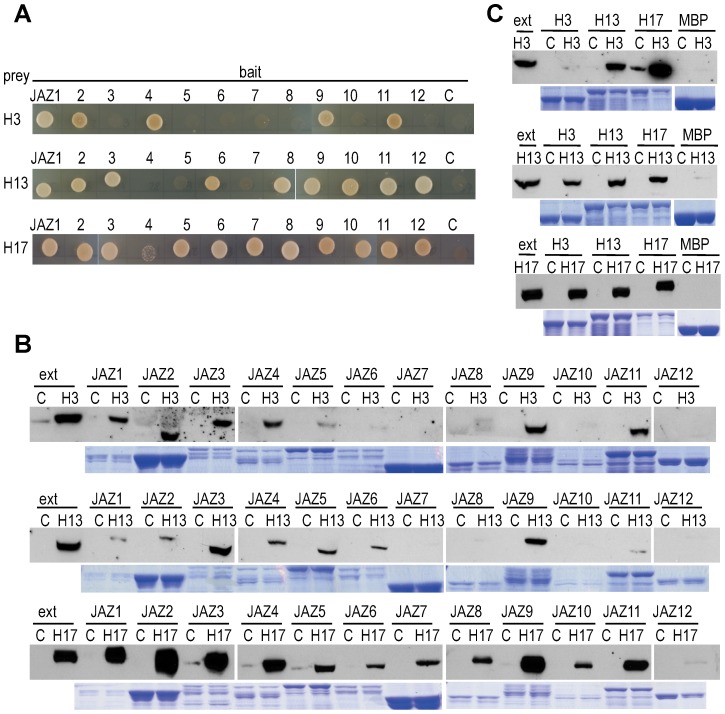
bHLH003, bHLH013 and bHLH017 interact with JAZ and can form homo and heterodimers. (A) Yeast cells co-transformed with the indicated combinations of JAZ-BD (pGBKT7) and bHLH-AD (pGADT7), grown for 3 days in medium lacking Ade, His, Leu and Trp to select for interactions. Numbers represent the number of JAZ protein (JAZ1 to JAZ12) and C represents the empty pGBKT7 vector containing only the BD. Transformation controls are shown in Figure S1. (B) Immunoblots with anti-HA antibody of the bHLH003-HA, bHLH013-HA and bHLH017-HA from transgenic plant extracts, pulled down by recombinant MBP-JAZ proteins expressed in *E.coli*. WT extracts were used as control (c). The two first lanes of each blot correspond to 40 µl of the input protein extract used for pull-down. Bellow each immunoblot, a coomassie stained gel shows the amount of recombinant MBP-fused protein used in each sample. (C) Immunoblots of pulled-down bHLH-HA proteins from transgenic plant extracts, by recombinant MBP-bHLH, presented as in (B) except that 30 µl from the total plant extract were loaded in the first lane of each gel. MBP controls are common for assays in (B) and (C) since both were done simultaneously.

To test if the JID domain in these bHLH TFs is functional, we first checked whether they interact with JAZ proteins in yeast two-hybrid assays. As shown in Figure 1A, bHLH017 interacts with all JAZ proteins (weakly with JAZ4), whereas bHLH013 interacts with most of them, but not with JAZ4, JAZ5 and JAZ7. bHLH003 showed a pattern of interaction more restricted, interacting only with JAZ1, JAZ2, JAZ4, JAZ9 and JAZ11.

To confirm these results we next tested the interactions between all three bHLHs and JAZ repressors in pull-down (PD) experiments. We generated transgenic Arabidopsis plants expressing bHLH-HA fusions and selected homozygous lines. Consistent with yeast results, bHLH017-HA was pulled-down from the transgenic extracts by all JAZ proteins, though with different efficiencies (Figure 1B). Similarly, bHLH003-HA was pulled-down by JAZ1, JAZ2, JAZ3, JAZ4, JAZ9 and JAZ11. Most MBP-JAZ proteins with the exception of JAZ7, JAZ8, JAZ10 and JAZ12 were able to pull-down bHLH013-HA. Thus, in general, PD analyses are consistent with Y2H assays and demonstrate that these bHLH proteins can interact with JAZ repressors *in vitro*.

To test if the interaction between JAZ proteins and bHLHs occur also *in vivo*, we performed Tandem Affinity Purification (TAP) of protein complexes in cultured PSB-D Arabidopsis cells using N- and C-terminal fusions of bHLH003, bHLH013 and bHLH017 with the TAP epitope as baits. As shown in Table 1 and Table S2, bHLH017-TAP allowed the co-purification of protein complexes that included several JAZ proteins (JAZ2, JAZ3, JAZ11 and JAZ12) and NINJA (although JAZ12 and NINJA were found only in one independent experiment; Table 1 and Table S2). Interestingly, these complexes also included bHLH003. Purification of bHLH003-TAP complexes identified bHLH013 and, conversely, TAP on bHLH013 rendered the isolation of bHLH003 (Table 1). Therefore, TAP tagging experiments revealed that these three bHLH proteins form heterodimers *in vivo* and interact with core components of JA signaling modules *in planta*.

**Table 1:** Proteins interacting with bHLH003, bHLH013 and bHLH017 in TAP tagging assays.

AT number	Protein name	bHLH003 (4)	bHLH013 (4)	bHLH017 (6)
AT4G16430	bHLH003	4	4	5
AT1G01260	bHLH013	4	4	
AT2G46510	bHLH017			6
AT1G74950	JAZ2			2
AT3G17860	JAZ3			2
AT3G43440	JAZ11			2
AT5G20900	JAZ12			1
AT4G28910	NINJA			1

The prey proteins identified in the TAP tagging assays using bHLH003-TAP, bHLH013-TAP and bHLH017-TAP as baits are listed in the left column. Numbers within parenthesis indicate the total number of TAP assays performed for each protein. The numbers in the table correspond to the number of positive results in the independent TAP assays. Half of the assays were performed with an N-terminal TAP fusion and the other half with a C-terminal TAP fusions expressed in Arabidopsis cells suspension cultures (PSB-D).

To further analyze the spectrum of heterodimeric interactions among the three bHLHs, we expressed and purified MBP-bHLH003, MBP-bHLH013 and MBP-bHLH017 proteins from *E.coli*, and tested them in PD experiments using extracts of the transgenic lines expressing HA fusions of the three bHLHs. Results of PD experiments (Figure 1C) confirmed the TAP-tagging data showing that all three proteins can form heterodimers and bHLH013 and bHLH017 can also form homodimers.

### Expression patterns of bHLH003, bHLH013 and bHLH017

**Figure 2:**
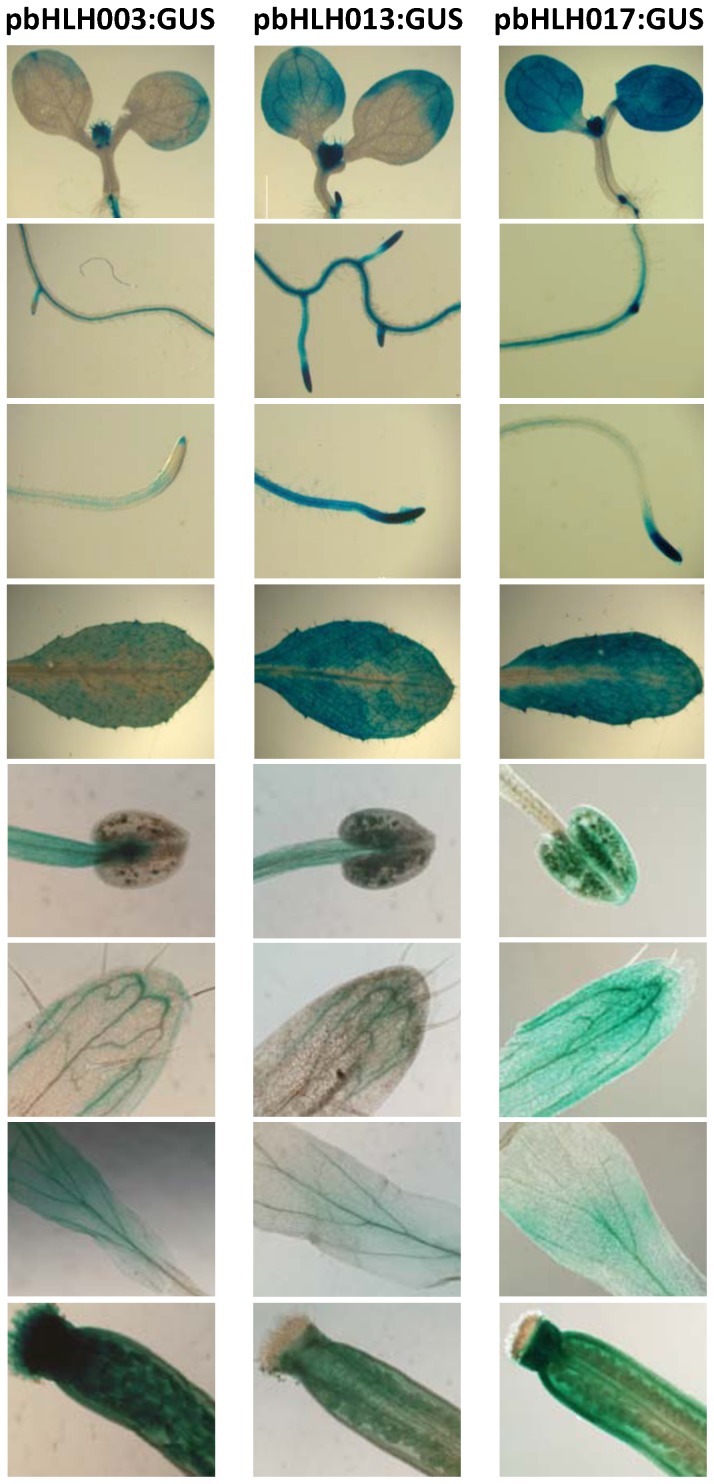
Expression patterns of bHLH003, bHLH013 and bHLH017 in Arabidopsis. Histochemical GUS staining of 6 days-old or adult Arabidopsis plants expressing the GUS reporter under the control of the bHLH003, bHLH013 and bHLH017 promoters. From upper to bottom panels, GUS activity was detected in cotyledon, roots, adult leaves, anthers, sepals, petals and pistil. GUS activity was detected in cotyledon and leaf, roots, petals, sepals, etc, etc.

To get an insight into the activity of these TFs in the plant we generated transgenic plants that express the β-glucuronidase (GUS) reporter under the control of the *bHLH003*, *bHLH013* or *bHLH017* promoters, and analyzed their tissue expression patterns in seedlings and adult plants. Consistent with microarray data at BAR (http://bar.utoronto.ca) all three genes were expressed quite ubiquitously. In 6 day-old seedlings, all three bHLHs were expressed in leaves, cotyledons and roots, predominantly in the vasculature and root tips (Figure 2). *bHLH017* expression was widespread over all aerial organs and *bHLH013* expression could be detected in all root tissues. Among the genes analyzed, *bHLH003* had the lowest expression levels in all tissues, but a strong GUS signal was detected in young emerging leaves.

In adult leaves, all three *bHLHs* showed the same expression patterns, however signal from *bHLH003* was weaker (Figure 2). GUS expression driven by all three promoters could be detected in all floral organs. In sepals, *bHLH017* showed a widespread expression while expression of *bHLH003* and *bHLH013* was confined to vasculature. In petals, expression of the three *bHLHs* was similar, and in reproductive organs *bHLH003* and *bHLH013* were more abundant in stamen filaments, while *bHLH017* is highly expressed in the anther. In all cases, GUS staining was detected in some pollen grains. In the pistil, all three *bHLH* genes are expressed across the ovary tissues while *bHLH003* is the only being expressed in stigma and ovules.

### bHLH003, bHLH013 and bHLH017 repress JA responses

Previous results suggest that bHLH003, bHLH013 and bHLH017 might play a redundant role in the regulation of JA-mediated responses. To test this hypothesis we obtained homozygous mutants from insertion lines available in NASC. RT-PCR analysis of gene expression confirmed that the homozygous T-DNA insertion lines in *bHLH003* (GK-301G05) and *bHLH017* (SAIL_536_F09) did not express full-length mRNAs (Figure S2), indicating that these lines should be Knock-Out mutants. However, T-DNA insertion in line GK-696A04 did not alter the expression of *bHLH013* (Figure S2). We also generated transgenic Arabidopsis lines constitutively and ectopically expressing *bHLH003*, *bHLH013* and *bHLH017* under the control of the CaMV 35S promoter. Thus, we analyzed the insertion lines of *bHLH003* and *bHLH017* and the OE lines of all three bHLHs for defects in JA-related phenotypes such as root- and aerial-growth inhibition, anthocyanin accumulation, chlorophyll loss and pathogen resistance.

**Figure 3:**
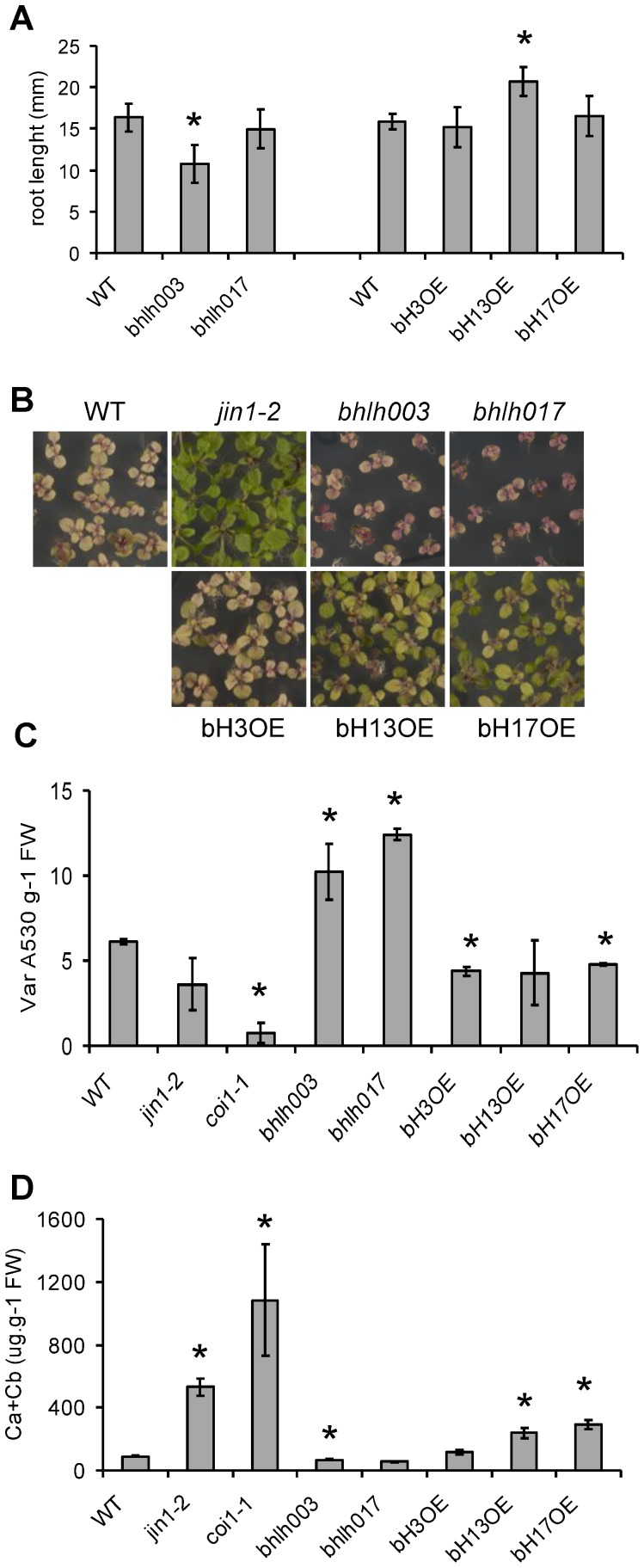
Responses to JA are affected in bHLH mutant and over-expression (OE) lines. (A) Root growth inhibition by JA (10 µM) of 8 days-old wild-type (WT), *bhlh003* and *bhlh017* mutant seedlings and OE lines of bHLH003, bHLH013 and bHLH017 grown in vertical MS plates. Values correspond to the average root growth betwen day 2 and day 8. Error bars represent standard deviations. Asterisks represents values that are significantly different statistically from WT applying a Student's t-test (p<0.01). (B) Phenotype of rosette leaves of 13 days-old seedlings grown in 50 µM JA-containing MS medium. (C) Anthocyanin accumulation and (D) Chlorophyll content in WT, *jin1-2*, *coi1-1*, *bhlh003* and *bhlh017* mutants and OE lines for bHLH003, bHLH0013 and bHLH017, grown in 50 µM JA for 13 days. Bars represent the average value of 3 pools of seedlings and error bars the standard deviation. Asterisks indicate statistically significant values compared to WT applying a Student's t-test (p<0.05).

JA-Ile treatment inhibits root and aerial plant growth. Root-length analysis of seedlings germinated and grown for 8 days in vertical plates containing 10 µM JA showed that root-growth inhibition by JA was significantly higher in *bhlh003* mutants than in WT plants (Figure 3A). In contrast, root growth of *bHLH013* over-expressing plants was less sensitive to JA.

Analysis of the aerial part of seedlings grown for 13 days in MS plates containing 50 µM JA revealed that *bhlh003* and *bhlh017* mutants were smaller and reddish (a symptom of higher accumulation of anthocyanins) than WT plants, suggesting that mutation of *bHLH003* or *bHLH017* might promote hypersensitivity to JA (Figure 3B). Consistent with this hypothesis, the 35S:*bHLH017* and 35S:*bHLH013* OE lines were bigger than WT plants and more similar to the JA-insensitive *jin1-2* mutant, indicating that constitutive activation of these two genes promote JA-insensitivity (Figure 3B). 35S:*bHLH003* also had a weak effect increasing aerial plant size.

Next, we analyzed anthocyanin accumulation in the mutants and OE lines. Exogenous JA treatment promotes accumulation of the pigments in WT plants. This increase is highly attenuated in *jin1-2* mutants and almost impaired in *coi1-1* (Figure 3B, 3C and Figure S3). Consistent with previous results, *bhlh003* and *bhlh017* mutants showed higher levels of anthocyanins than WT, whereas transgenic plants overexpressing any of the three bHLH TFs accumulated lower levels than WT, similar to *jin1-2* (Figure 3C).

JA treatment also produces a reduction of chlorophylls in plant leaves, which depends on *jin1-2* and *coi1-1* (Figure 3D). In contrast, transgenic plants overexpressing *bHLH013* or *bHLH017* accumulated higher levels of chlorophylls than WT plants, further suggesting that these two TFs antagonize this JA effect. Consistent with this, levels of chlorophylls in *bhlh003* plants are reduced. Levels in *bhlh017* and 35S:*bHLH013* were comparable to WT (Figure 3D and Figure S3).

**Figure 4:**
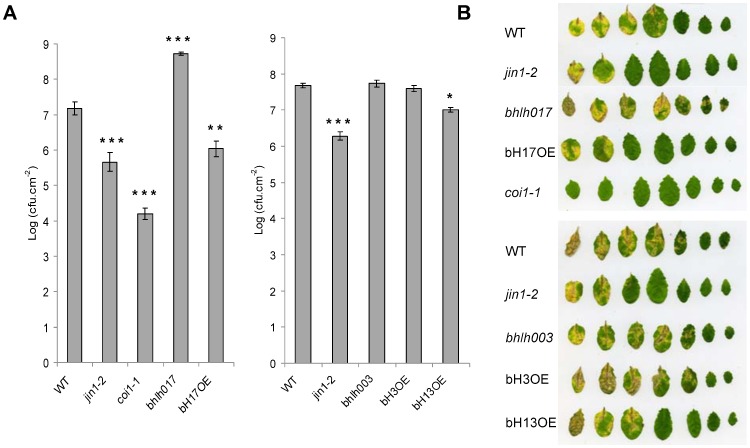
Resistance of bHLH mutants and OE lines to the bacterial pathogen Pto DC3000. (A) Growth of *Pseudomonas syringae* pv. *tomato* (*Pto*) DC3000 on WT Arabidopsis plants, *jin1-2*, *coi1-1*, *bhlh003* and *bhlh017* mutants and OE lines for bHLH003, bHLH0013 and bHLH017 3 days after spray inoculation. Bacterial counts are expressed as log (cfu.cm^−2^). Error bars indicate SE. The results are representative of two independent experiments. Asterisks indicate statistically significant differences compared with WT (Student's t test: *, P<0.05; **, P<0.05; and ***, P<0.001). (B) Disease symptoms in leaves of WT, mutants and OE lines. Pictures of detached leaves were taken 4 days after inoculation.

Some strains of Pseudomonas, such as *Pseudomonas syringae* pv. *tomato* (*Pto*) DC3000, produce Coronatine (COR), a bacterial phytotoxin that functionally mimics JA-Ile [13], [50]. COR activates the JA-pathway, which counteracts SA-dependent defenses against the bacteria [51], [52]. Thus, JA-insensitive mutants such as *jin1-2* or *coi1-1* are more resistant to Pto DC3000 than WT plants ([51] and Figure 4). The *bhlh017* mutant showed a strong susceptibility, increasing bacterial growth over one log compared to WT. Consistently, OE of bHLH017 or bHLH013 increased resistance reducing bacterial growth by one or half a log, respectively. The mutant or OE lines of bHLH003 did not show significant differences with WT plants.

Altogether, results from all phenotypic analyses are consistent with a role of bHLH003, bHLH013 and bHLH017 as repressors of different aspects of the JA function in seedling development and in plant defense.

### DNA-binding specificity of bHLH003, bHLH013 and bHLH017

Results described above suggest that bHLH003, bHLH013 and bHLH017 have an antagonistic effect on the JA pathway to that of MYC2, MYC3 and MYC4 [28]. TAP tagging results obtained in this work for bHLH003, bHLH013 and bHLH017, and by Fernández-Calvo et al. [28], for MYC2, MYC3 and MYC4, suggest that these two groups of TFs do not interact with each other *in vivo*. Direct testing of bHLH017 interaction with MYC2, MYC3 and MYC4 in co-immunoprecipitation assays supports this conclusion [28]. This has been also recently confirmed by Song et al. [49]. Therefore, we hypothesize that the antagonistic activity of these two groups of TFs might occur by competition for their binding sites. To test this hypothesis we determined the subcellular localization and the DNA-binding specificity of bHLH003, bHLH013 and bHLH017 and compared it with that of MYC2.

**Figure 5:**
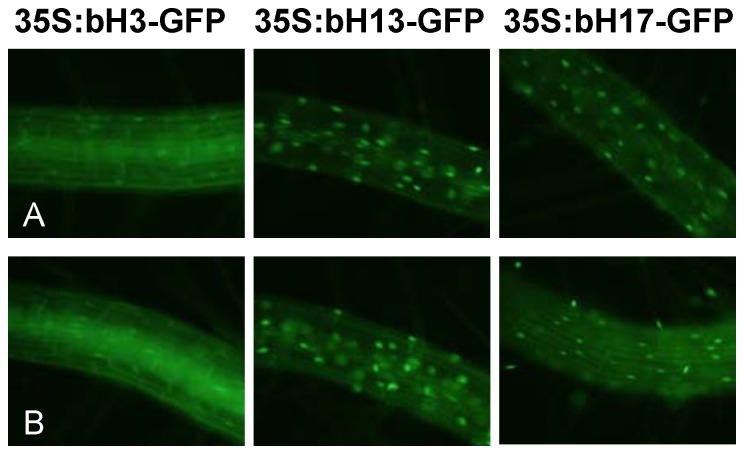
Sub-cellular localization of bHLH003, bHLH013 and bHLH017. Microscopy images of GFP signal detected in root cells of transgenic Arabidopsis transgenic plants over-expressing bHLH003-GFP, bHLH013-GFP or bHLH017-GFP and grown for ten days in media supplemented (A) or not (B) with 50 µM JA for 3 h.

To assess their subcellular localization we generated transgenic Arabidopsis plants expressing GFP fusions of these three TFs under the control of the 35S CaMV promoter. As shown in Figure 5, all three TFs showed a clear nuclear fluorescent signal in the root cells of the transgenic lines analyzed, confirming that they localize in the nucleus, as expected for a TF. Nevertheless, some background signal could be also detected in the cytoplasm, being more evident for bHLH003. We could not detect changes in the sub-cellular localization of any of the three bHLH proteins analyzed upon JA treatment (Figure 5B).

**Figure 6:**
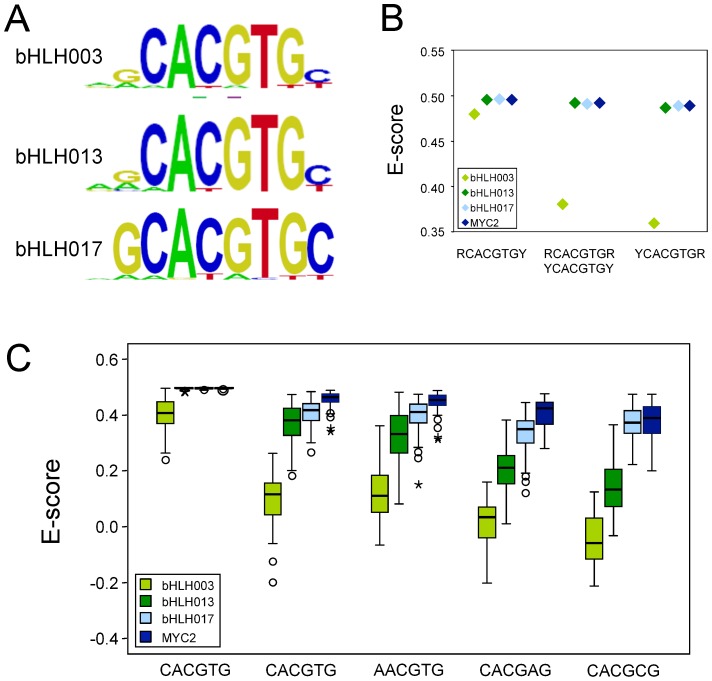
DNA-binding specificity of bHLH003, bHLH013 and bHLH017. (A) Position weight matrix (PWM) representation of the three top scoring 8-mers obtained in “seed-and-wobble” algorithm corresponding to bHLH003, bHLH013 and bHLH017. All three proteins showed highest binding affinity to a canonical G-box (CACGTG). (B) Median enrichment scores (E-scores) of the all the possible G-containing 8mers flanked by purine (R) and/or pyrimidine (Y) nucleotides recognized by the three bHLH proteins tested. bHLH003 showed a strong dependence for purine at 5′-end and pyrimidine at 3′-end. We included for comparison data corresponding to MYC2 previously described [53]. (C) Box-plot of E-scores of G-related variants. Boxes represent quartiles 25% to 75%, and black line represents the median of the distribution (quartile 50%). Bars indicate quartiles 1 to 25% (above) and 75 to 100% (below), and dots denote outliers of the distribution. Data corresponding to MYC2 were previously described [53].

We next determined the DNA-binding specificity of bHLH003, bHLH013, and bHLH017 *in vitro* using a protein binding microarray (PBM) assay [53]. The three proteins recognized with the highest affinity a perfect G-box (CACGTG; Figure 6A), similarly to that observed for MYC2, MYC3 and MYC4 using the same strategy [28], [53]. Analysis of the affinity for all possible 8-mers containing the G-box showed that the three proteins have a preference for purine at 5′-end and pyrimidine at 3′-end flanking the G-box (Figure 6B). However, whereas bHLH013 and bHLH017 (as well as MYC2) recognized with high affinity all the G-box-containing 8mers, binding of bHLH003 was almost exclusive for DNA sequences flanked by purine and pyrimidine at 5′ and 3′ ends, respectively (Figure 6B).

In previous works, we determined that MYC2, MYC3 and MYC4 proteins recognize with high affinity other DNA-elements related to the G-box, referred to as T/G, G-related, G/A and G/C elements [28], [53]. The analysis of binding to the DNA probes containing these variants revealed a strikingly similar pattern of affinities to the different variants for bHLH017 and MYC2 (Figure 6C), suggesting that they might bind similar cis-elements *in vivo*. bHLH013 showed also a clear binding to the MYC2-recognized elements, although with lower affinity than MYC2. bHLH003, however, seems to have a strict requirement for a perfect G-box flanked by purine and pyrimidine as mentioned above. Altogether, these results support that the antagonistic activity of these two groups of TFs might be achieved by competition for their binding sites.

### bHLH003, bHLH013 and bHLH017 are transcriptional repressors

**Figure 7:**
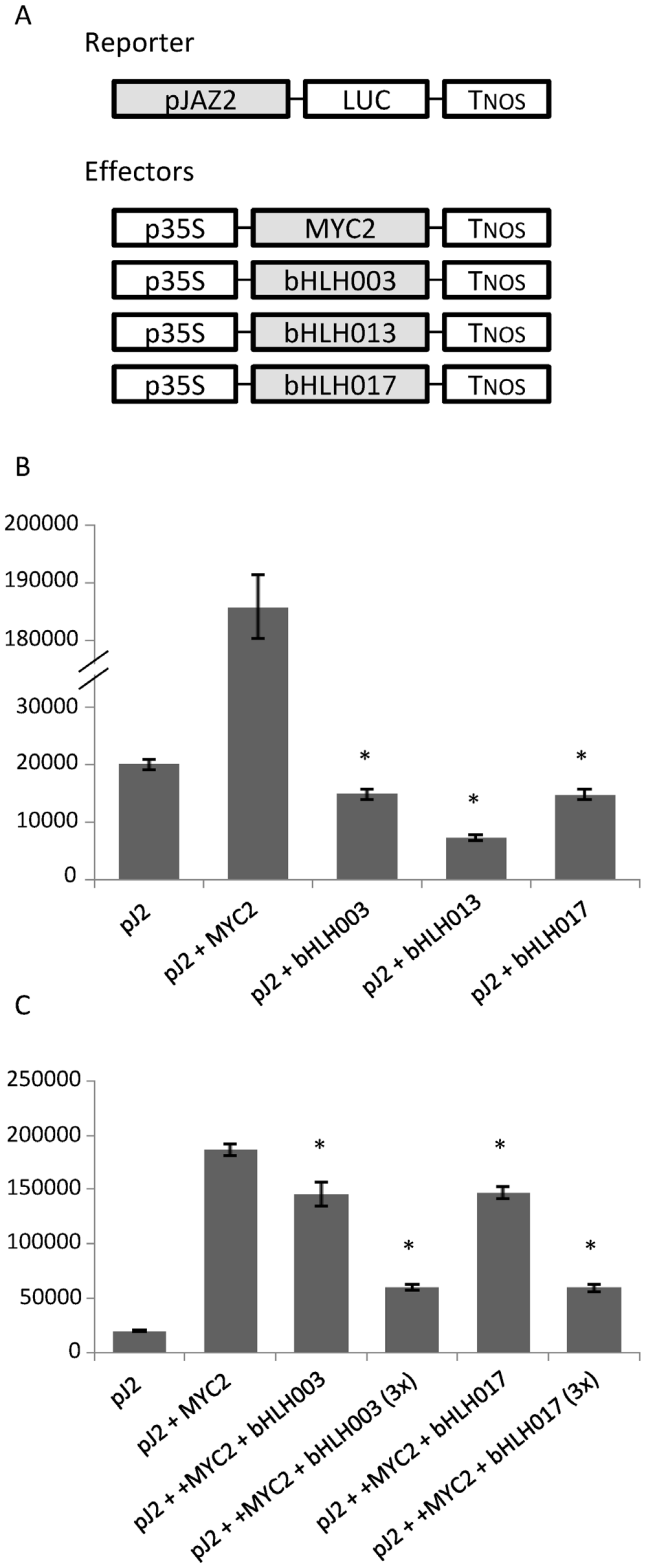
bHLH003, bHLH013 and bHLH017 are transcriptional repressors. (A) Schematic representation of reporter and effector constructs used in transient expression experiments *in Nicotiana benthamiana*. The reporter is the fusion of the JAZ2 promoter to firefly LUC coding sequence. MYC2, bHLH003, bHLH013 and bHLH017 genes expressed under the CaMV 35S promoter were used as effectors. (B) Induction or repression of *pJAZ2:LUC* reporter by MYC2, bHLH003, bHLH013 and bHLH017. Error bars indicate the SE from 16 replicates. (C) Effect of bHLH003 or bHLH017 expression on the MYC2 transactivation activity of *pJAZ2:LUC*. Error bars indicate the SE of results of 16 replicates. Asterisks represent p<0,05 in Students t-test.

To test the repressor activity of bHLH003, bHLH013 and bHLH017 we performed transient expression assays in *Nicotiana benthamiana* leaves. As effectors, we obtained 35S:HA-fusions of all three bHLHs and also MYC2 as a control for transcriptional activator. The promoter region (2 kb) of *JAZ2* fused to luciferase was used as reporter (Figure 7A). As shown in Figure 7B, co-expression of *pJAZ2:Luc* with 35S:MYC2-HA promoted an increase of the reporter activity, consistent with the function of MYC2 as a transcriptional activator. In contrast, co-expression of *pJAZ2:Luc* with any of the three bHLHs constructs reduced reporter's activity, indicating that these three TFs are transcriptional repressors (Figure 7B). Moreover, co-expression of the reporter with a combination of 35S:MYC2-HA and 35S:bHLH003-HA or 35S:bHLH017-HA had an additive effect reducing the transactivation capacity of MYC2 (Figure 7C). These results support that these bHLH TFs compete with MYC2 for their DNA-binding site *in vivo*.

### Expression of bHLH017 is induced by JA and depends on MYC2

**Figure 8:**
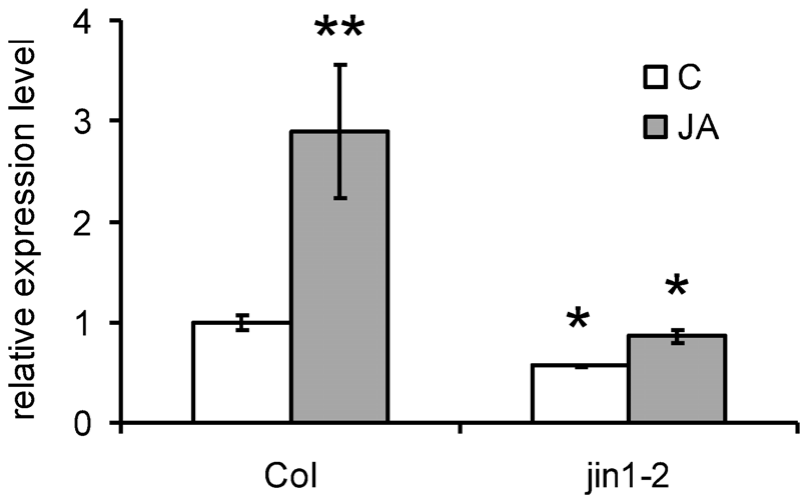
bHLH0017 induction by JA is reduced in *myc2* mutants. Quantitative real-time PCR of bHLH17 expression in WT plants or the *myc2* mutant allele *jin1-2* treated (or not) with 50 µM JA for 6 h. The measurements correspond to the average of three technical replicates and are relative to untreated WT. ACTIN8 expression was used as internal control. Error bars represent standard deviation. Asterisks indicate statistically significant differences compared to WT (Student's t test: * P<0,5 and ** P<0,005).

According to public databases (http://bar.utoronto.ca), *bHLH017* expression is induced by JA. To confirm it, we analyzed its mRNA accumulation by qRT-PCR in JA-treated WT plants and *jin1-2* mutants. As shown in Figure 8, *bHLH017* mRNA is induced by JA in WT plants, but this induction is greatly reduced in *jin1-2* mutants. This suggests that *bHLH017* expression is regulated by MYC2, and therefore, that activation of MYC2-dependent transcription triggers a negative feed-back regulation by bHLH017.

## Discussion

The current view of how JA responses are activated involves the de-repression of positive activators (TFs) of gene expression by protein degradation of JAZ repressors upon hormone binding to its receptor [5], [32]. In this work, we found that JAZ proteins also interact with negative regulators of gene expression, which contribute to repress hormone responses. Therefore, our results support that the JA output depends on the equilibrium between activator and repressor TFs.

### New JA-signaling modules

Identification of the JAZ-Interaction Domain (JID) of MYC2, MYC3 and MYC4 and BLAST searches with this sequence suggested new JAZ targets [28]. A combination of protein-protein interaction techniques used in this work (Y2H, PD and TAP tagging) has confirmed that JAZ proteins interact with bHLH003, bHLH013 and bHLH017 *in vivo* and *in vitro*, uncovering new JA-signaling modules. These results are in line with those very recently described by Song et al., [49], which also demonstrated that JAZ proteins repress these bHLHs. TAP tagging and PD results also showed that these three TFs can form homo- and hetero-dimers, thus indicating that a variety of combinations among these three TFs and JAZ proteins can be expected, broadening the regulatory possibilities of these signaling modules. This resembles the situation described for MYC2, MYC3 and MYC4, which can also homo- and hetero-dimerize to form distinct regulatory complexes [28]. Interestingly, based on Y2H, PD and TAP tagging results (this work and [28], [49]) bHLH003, bHLH013 and bHLH017 do not seem to hetero-dimerize with MYC2, MYC3 or MYC4, suggesting that these two groups of TFs represent two separate clades that do not interact with each other and form distinct JA-signaling modules.

### A new mechanism of negative regulation of JA responses

bHLH003, bHLH013 and bHLH017 are nuclear proteins with a DNA-binding specificity strikingly similar to that of MYC2, MYC3 and MYC4, and with similar tissue expression patterns, suggesting that they might regulate similar, or at least overlapping, sets of genes. However, sequence analysis of bHLH003, bHLH013 and bHLH017 fail to identify an activation domain conserved with MYC2, MYC3 and MYC4, which suggested that they might behave as repressors rather than activators [28], [47]. Consistently, transient expression experiments in *Nicotiana benthamiana* and phenotypic analyses of mutants and transgenic OE lines demonstrated that these three bHLHs are transcriptional repressors, regulating JA responses in the opposite way to MYC2, MYC3 and MYC4 (this work and [28], [47]–[49]).

The fact that these two clades do not seem to form heterodimers suggests that competition for DNA-binding might mechanistically explain their functional antagonism. Supporting this hypothesis, transcriptional activity of MYC2 can be repressed by bHLH003 or bHLH017 in transient experiments in *Nicotiana benthamiana* leaves and Arabidopsis protoplasts (this work and [47], [49]).

**Figure 9:**
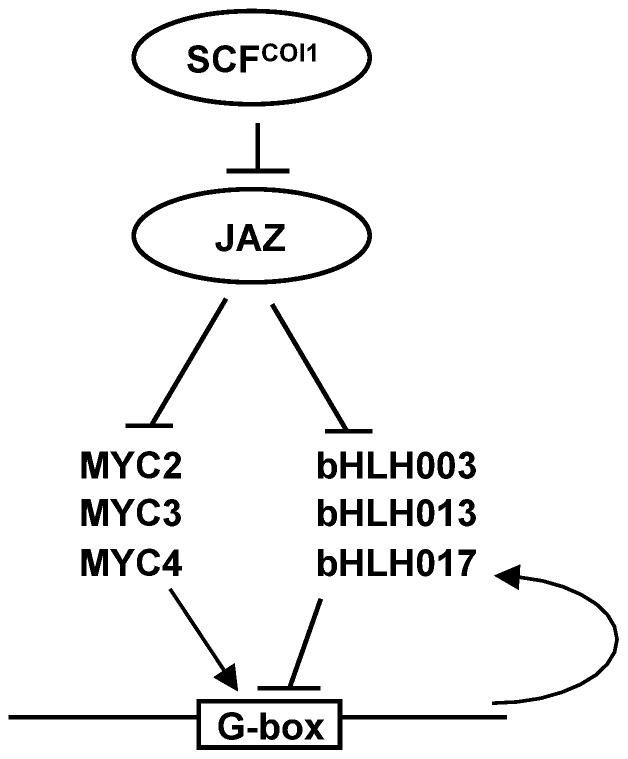
Schematic model of the role of bHLHs within the JA signaling pathway. In the absence of JA-Ile, JAZ repressors are stable and interact with MYC2, MYC3 and MYC4 as well as bHLH003, bHLH013 and bHLH017. JAZs are part of a repression complex that comprises NINJA and TOPLESS (not shown). Upon a stimulus, hormone is perceived by the COI1/JAZ co-receptor and JAZ proteins are targeted for degradation by the proteasome. Once released from JAZ, MYC2, MYC3 and MYC4 will activate transcription, whereas bHLH003, bHLH013 and bHLH017 will repress it. Both sets of proteins (MYCs and bHLHs) compete for the G-box and, therefore, the output response will depend on the balance of activity between these two sets of TFs. Moreover, bHLH017 expression is activated by MYC2, therefore increasing the effective repressor over time and further contributing to reduce transcriptional activation.

The lack of a repressor domain, such as the EAR present in many TFs [21] suggest that bHLH003, bHLH013 and bHLH017 may exert their repressive role through two independent mechanisms: i) by competition for the DNA-binding sites and interference with the transcriptional activators, and ii) by their interaction with JAZ repressors and recruitment of TPL and TPR co-repressors through the adaptor protein NINJA [22]. Thus, in our current view (Figure 9), bHLH003, bHLH013 and bHLH017 may form repression complexes with JAZ-NINJA-TPL, similar to those described for MYC2, MYC3 and MYC4. It should be noted that in the absence of the activation signal (JA-Ile) MYC2, MYC3 and MYC4 complexes with JAZ-NINJA-TPL are also repressor complexes because their DNA-binding specificity determines the genes to be repressed by TPL/TPRs. In the presence of JA-Ile, degradation of JAZ separates the TFs from the NINJA-TPL/TPRs co-repressors. In the case of MYC2, MYC3 and MYC4, which have an activation domain, the release of the TFs will allow the activation of their target genes. However, in the case of bHLH003, bHLH013 and bHLH017, the lack of an activation domain would prevent any transcriptional activation and would reduce the efficiency of MYC2, MYC3 and MYC4 due to competition for binding to their *cis*-regulatory elements. Moreover, since expression of bHLH017 is dependent on MYC2, activation of MYC2 will increase the concentration of bHLH017, therefore increasing competition for their DNA-binding targets and reducing the activity of the positive transcriptional activators.

It is becoming evident that repression of the JA pathway is extremely important for the cell to prevent harmful responses and to fine-tune activation spatio-temporaly. Several repression mechanisms have been described recently. Thus, transcriptional activation of the JAZ genes contributes to re-establish the repressor complexes [10], [15], [22]. Repression is also potentiated by the expression of JAZ repressor forms resistant to degradation, i.e. truncated forms of JAZ (JAZΔJas [17], [37]–[39]) and JAZ8 [23]. The identification of these new repressor TFs adds a new example to these mechanisms of repression operating by TF competition for their *cis*-regulatory elements in the promoters of JA-regulated genes.

## Materials and Methods

### Plant material and growth conditions

*Arabidopsis thaliana* Columbia (Col-0) is the genetic background of wild-type and transgenic lines used throughout the work. Seedlings were grown in Murashige and Skoog medium (Sigma-Aldrich) at 21°C under a 16-h-light/8-h-dark cycle. The T-DNA insertion lines *bhlh003* (GK-301G05), *bhlh013* (GK-696A04) and *bhlh017* (SAIL_536_F09) were obtained from the Nottingham Arabidopsis Stock Centre (NASC), *myc2/jin1-2* was previously described [26] and *coi1-1* was kindly provided by J. Turner.

To generate transgenic plants expressing bHLH003, bHLH013 or bHLH017 in Col-0 background, full-length coding sequences carrying or not the stop codon were amplified with Expand High Fidelity polymerase (Roche) using Gateway-compatible primers (Sup Table S1).

PCR products were cloned into pDONR207 using the Gateway system (Invitrogen), and those without stop codon transferred to pGWB5 and pGWB14 and sequence verified. Agrobacterium strain GV3101, containing these constructs, was used to transform Col-0 plants by floral dipping [54]. Homozygous and independent lines of 35S:bHLH003-GFP, 35S:bHLH003-HA, 35S:bHLH013-GFP, 35S:bHLH013-HA, 35S:bHLH017-GFP and 35S:bHLH017-HA were selected and used for further analysis.

### Sub-cellular localization

Fluorescence of Arabidopsis 35S:bHLH003-GFP, 35S:bHLH013-GFP and 35S:bHLH017-GFP transgenic seedlings treated or untreated with 50 µM JA during 3 h was visualized by a Leica DMR UV/VIS microscope under UV light.

### Root measurements

Root growth from day 2 to day 8 after germination was measured on 10 to 15 seedlings grown in vertical MS plates, in the presence or absence of 10 µM JA (Sigma-Aldrich). At least three independent experiments were performed. Values represent mean ± sd. Student's t-test was applied.

### Anthocyanin and chlorophyll quantification

Seedlings were grown for 10 days in MS medium or 13 days in media supplemented with 50 µM JA. The aerial part of 6 to 12 seedlings was pooled for each replicate. Three independent replicates were collected from different plates. Anthocyanin quantification was performed as described by Swain and Hillis, [55]. Values represent mean ± s.d and Student's t-test was applied. The experiment was repeated three times with similar results.

Six to 10 seedlings from the same plates were pooled for chlorophyll measurements. Acetone 80% (V/V) was used for extraction and A_645_ and A_663_ was measured in a spectrophotometer. Data analysis was done according to Arnon, [56]. Values represent mean ± s.d. The experiment was repeated three times.

### Yeast two-hybrid assays

Full-length bHLH003, bHLH013 or bHLH017 coding sequences carrying a stop codon were recombined from pDORN207 into pGADT7 (Gal4 AD). JAZ sequences cloned into pGBKT7 (Gal4 BD) were previously described in Chini et al., [57]. To assess protein interactions, the corresponding plasmids were co-transformed into yeast AH109 cells following standard heat shock protocols. The method used for Y2H assays was previously described [28], [57]. Colonies from the co-transformed plates were collected and resuspended in minimal medium. A drop of each experiment was plated in medium lacking Leu and Trp to select for co-transformation and in medium lacking Ade, His, Leu, and Trp to select for interaction. Photos were taken after 3 days incubation at 30°C. Empty gateway vectors pGADT7 and pGBKT7 were used for co-transformation as negative controls.

### Protein extracts and pull-down assays

MBP-JAZ fusion proteins were generated as previously described [11], [57] and MBP-bHLH fusions were transferred from the pDONR207 into the pDEST-TH1 [58] by recombination (Gateway, Invitrogen). Recombinant protein purification from E. coli was performed according to Fonseca and Solano [59]. Ten days-old Arabidopsis wild-type seedlings and transgenic lines expressing 35S:bHLH003-HA, 35S:bHLH013-HA and 35S:bHLH017-HA were ground in liquid nitrogen and homogenized in extraction buffer containing 50 mM Tris-HCl, pH 7.4, 80 mM NaCl, 10% glycerol, 0.1% Tween-20, 1 mM DTT, 1 mM phenylmethylsulphonyl fluoride, 50 µM MG132 (Sigma-Aldrich) and complete protease inhibitor (Roche) and used in pull-down assays [59].

### TAP-tagging purification

Cloning of transgenes encoding tag fusions under control of the constitutive cauliflower tobacco mosaic virus 35S promoter and transformation of Arabidopsis cell suspension cultures were performed as previously described using the oligonucleotides listed in Sup Table S1 [60]. TAP of protein complexes was done using the GS tag [61] followed by protein precipitation and separation, according to Van Leene et al. [62]. The protocols of proteolysis and peptide isolation, acquisition of mass spectra by a 4800 Proteomics Analyzer (Applied Biosystems) and MS-based protein homology identification based on the TAIR genomic database are described in Van Leene et al. [63]. Experimental background proteins were subtracted based on ∼40 TAP experiments on wild-type cultures and cultures expressing TAP-tagged mock proteins GUS, red fluorescent protein, and GFP [63].

### Determination of DNA-binding motifs

DNA-binding specificities of bHLH003, bHLH013 and bHLH017 were determined using protein binding microarrays (PBM) as described by Godoy et al. [53], from soluble protein extracts. Extracts were obtained from 25 mL induced *E. coli* cultures containing the translational fusions to MBP. Pelleted cells were resuspended in 1 mL of 1x binding buffer, sonicated (2×30 s) and centrifuged twice to obtain cleared extracts of soluble proteins as in Godoy et al., [53]. The extract was adjusted to 175 µl containing 2% milk and 0.89 µg of denatured salmon sperm DNA. Synthesis of double-stranded microarrays, protein incubations and immunological detections of DNA-protein complexes were as described [53], [64].

### Promoter fusions and GUS staining

bHLH003, bHHL013 and bHLH017 promoter regions of 1512 bp, 2121 bp and 2574 bp from the ATG, respectively, were amplified by combining the oligonucleotides listed in Table S1, cloned in pDONR207 (bHLH003 or bHLH013) or in pENTR/D-TOPO (Invitrogen, bHLH017) and transferred to pGWB3, to drive GUS expression. Agrobacterium GV3101 strain was transformed with these constructs and used to transform Arabidopsis by floral dipping [54].

Six days-old seedlings or adult plant tissues from several transgenic lines were stained for GUS activity. Samples were placed in staining solution containing 50 mM phosphate buffer, pH 7, 0.1% (v/v) Triton X-100 (Sigma-Aldrich), 2 mM 5-bromo-4-chloro-3-indolyl b-D glucuronic acid (X-Gluc, Glycosynth), 1 mM potassium-ferrocyanide (Sigma-Aldrich), and 1 mM potassium-ferricyanide (Sigma-Aldrich) and incubated at 37°C overnight. After staining, the tissue was soaked several times in 75% ethanol and kept in 5% glycerol until being photographed with a Leica DMR UV/VIS microscope (anthers, sepals, petals and pistils) or with a digital NIKON D1-x camera (seedlings and adult leaves).

### Luciferase assay

Transcriptional activity of bHLH003, bHLH013 or bHLH017 and competition with MYC2 for their binding sites in the JAZ promoters was measured using the promoter of JAZ2 (2 Kb) fused to a Luciferase reporter gene cloned into pGWB435. Leaves of *N. benthamiana* were transiently infiltrated with Agrobacterium strains bearing the *pJAZ2:LUC* and *35S:MYC2-HA*, *35S:bHLH0003-HA*; *35S:bHLH013-HA* or *35S:bHLH017-HA* constructs. All combinations included the silencing suppressor p19. 24 hours after agroinfiltration, 1 cm discs were collected from the leaves with the aid of a cylindrical borer and carefully transferred, the abaxial side upwards, to 96 well microplates filled with 175 µl of H_2_O and 25 µl of D-Luciferin substrate (0.1 mg/ml; Sigma #L9504). One disc was used per well and at least 16 disc replicates per sample. Levels of Luciferase activity were measured every hour, for a total of 48 hours, using the LB 960 Microplate Luminometer (Berthold) which operates through the Windows® PC MikroWin 2000 software.

### Quantitative real time PCR (QPCR)

Seedlings untreated or treated with 50 µM JA for 6 h were harvested for RNA extraction with the Trizol reagent (Invitrogen). After DNase I digestion and cleanup by RNeasy mini kit (Qiagen) to completely remove genomic DNA, one µg of total RNA was used for reverse transcription (High-Capacity cDNA Reverse Transcription kit, Applied Biosystems). *bHLH0017* and *ACTIN8* (used as internal control) were amplified with the oligos listed in Table S1, with Power SYBR Green mix (Applied Biosystems) in a 7300 Real Time PCR system (Applied Biosystems). Data analysis was done using three technical replicates from one biological sample and experiment was independently repeated with similar results.

### Infection assays with *Pseudomonas syringae*

*Pseudomonas syringae* pv *tomato* (*Pto*) DC3000 growth assays in Arabidopsis were performed by spray inoculation as previously described in Fernandez-Calvo et al, [28]. Briefly, overnight bacterial cultures were pelleted and resuspended in sterile 10 mM MgCl_2_. Three- to four week-old plants were sprayed with a bacterial suspension containing 10^8^ (colony-forming units)/mL bacteria (OD_600_ = 0.2) with 0.04% Silwet L-77. Leaf discs were harvested after 2 days and ground in 10 mM MgCl_2_. Population counts were performed at 2 days after infiltration. In both cases, serial dilutions of leaf extracts were plated on LB agar with appropriate antibiotics. Data points represent the average of four replicates, each containing two leaf discs from different plants. Error bars indicate SE. These experiments were repeated with similar results, and representative results are shown. Pictures of disease symptoms 4 days after inoculation on analyzed genotypes were taken with a digital NIKON D1-x.

## Supporting Information

Figure S1 **Co-transformation controls of Y2H assays.** Yeast growth control of experiment shown in Figure 1A. Yeast cells co-transformed with the indicated combinations of JAZ-BD (pGBKT7) and bHLH-AD (pGADT7), grown for 3 days in medium lacking Leu and Trp to select for co-transformation. Numbers represent the number of JAZ protein (JAZ1 to JAZ12) and C represents the empty pGBKT7 vector containing only the BD.
(TIFF)

Figure S2 **T-DNA insertion lines for bHLH003, bHLH013 and bHLH017.** (**A**) Representation of T-DNA insertions (black triangles) in bHLH003, bHLH013 and bHLH017 genes. The white bar represents the coding sequence where the bHLH domain is shown in grey. Pairs of oligonucleotides used for cDNA amplification are shown as arrows. (**B**) PCR products generated with the oligonucleotide pairs indicated above, in WT and mutant T-DNA insertion homozygous lines. Oligonucleotide sequence is listed in Table S1.
(TIFF)

Figure S3 **Anthocyanin and chlorophyll quantification in bHLH003, bHLH013 and bHLH017 mutants and OE lines in basal conditions.** Anthocyanin accumulation (**A**) and chlorophyll content (**B**) in 10 days-old WT seedlings, *jin1-2*, *coi1-1*, *bhlh003* and *bhlh017* mutants and OE lines for bHLH003, bHLH0013 and bHLH017. Bars represent the average of three pools of seedlings and error bars the standard deviation. Differences are not statistically significant (Student's T-test).
(TIFF)

Table S1 **Oligonucleotides used for PCR reactions described in Material and Methods section.**
(TIFF)

Table S2 **Protein Identification details obtained with the 4800 MALDI TOF/TOF Proteomics analyzer (AB SCIEX) and the GPS explorer v3.6 (AB SCIEX) software package combined with search engine Mascot version 2.2 (Matrix Science) and database TAIR.** Column headers for Protein and Peptide data are explained below.
(XLSX)
