# Supplementary figures and images for: bHLH003, bHLH013 and bHLH017 Are New Targets of JAZ Repressors Negatively Regulating JA Responses
Source: PLoS One. 2014 Jan 23;9(1):e86182. doi: 10.1371/journal.pone.0086182 (PMC3900477; doi:10.1371/journal.pone.0086182)

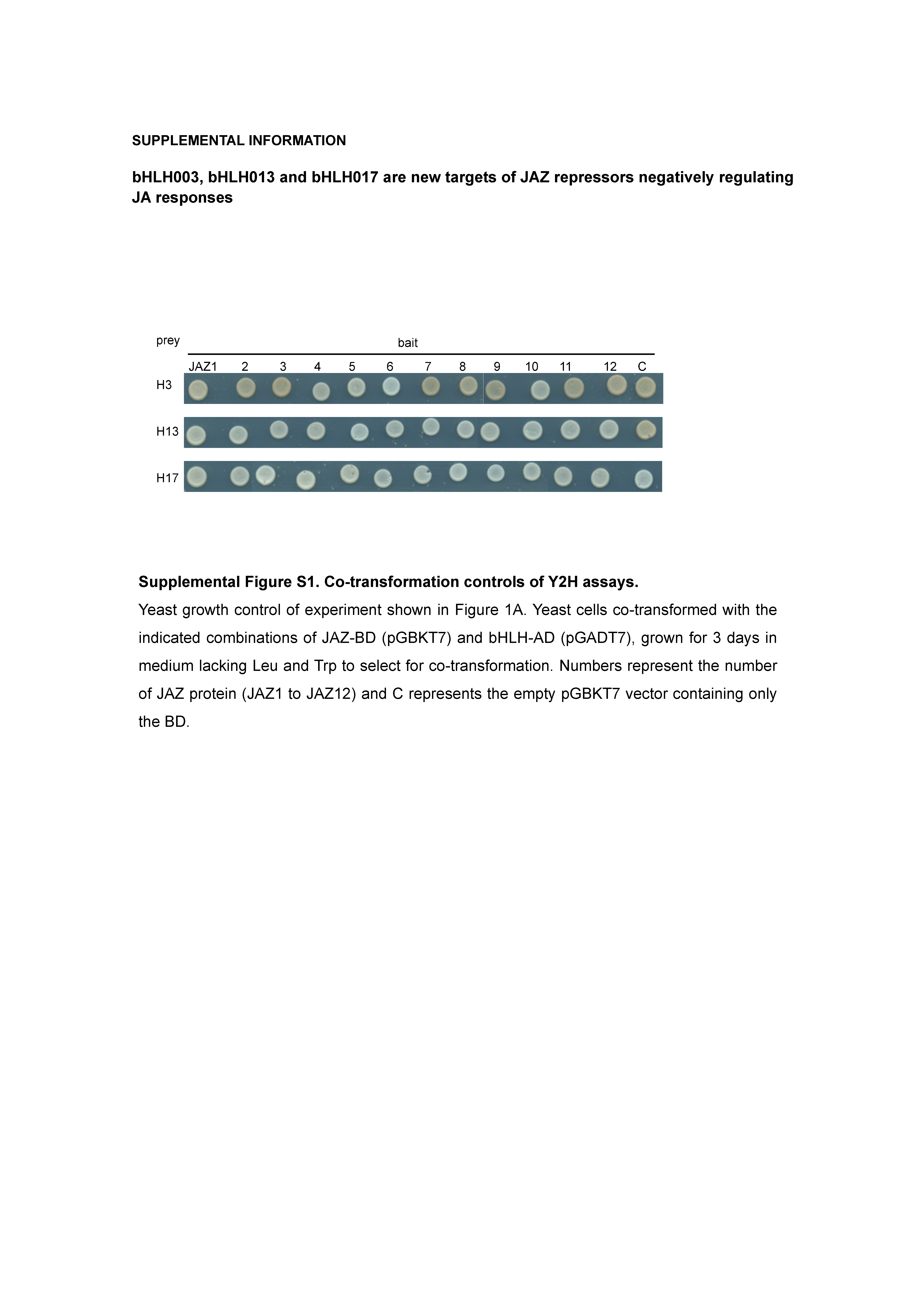

Supplement: Figure S1 — Co-transformation controls of Y2H assays. Yeast growth control of experiment shown in Figure 1A. Yeast cells co-transformed with the indicated combinations of JAZ-BD (pGBKT7) and bHLH-AD (pGADT7), grown for 3 days in medium lacking Leu and Trp to select for co-transformation. Numbers represent the number of JAZ protein (JAZ1 to JAZ12) and C represents the empty pGBKT7 vector containing only the BD. (TIFF) [file pone.0086182.s001.tiff]

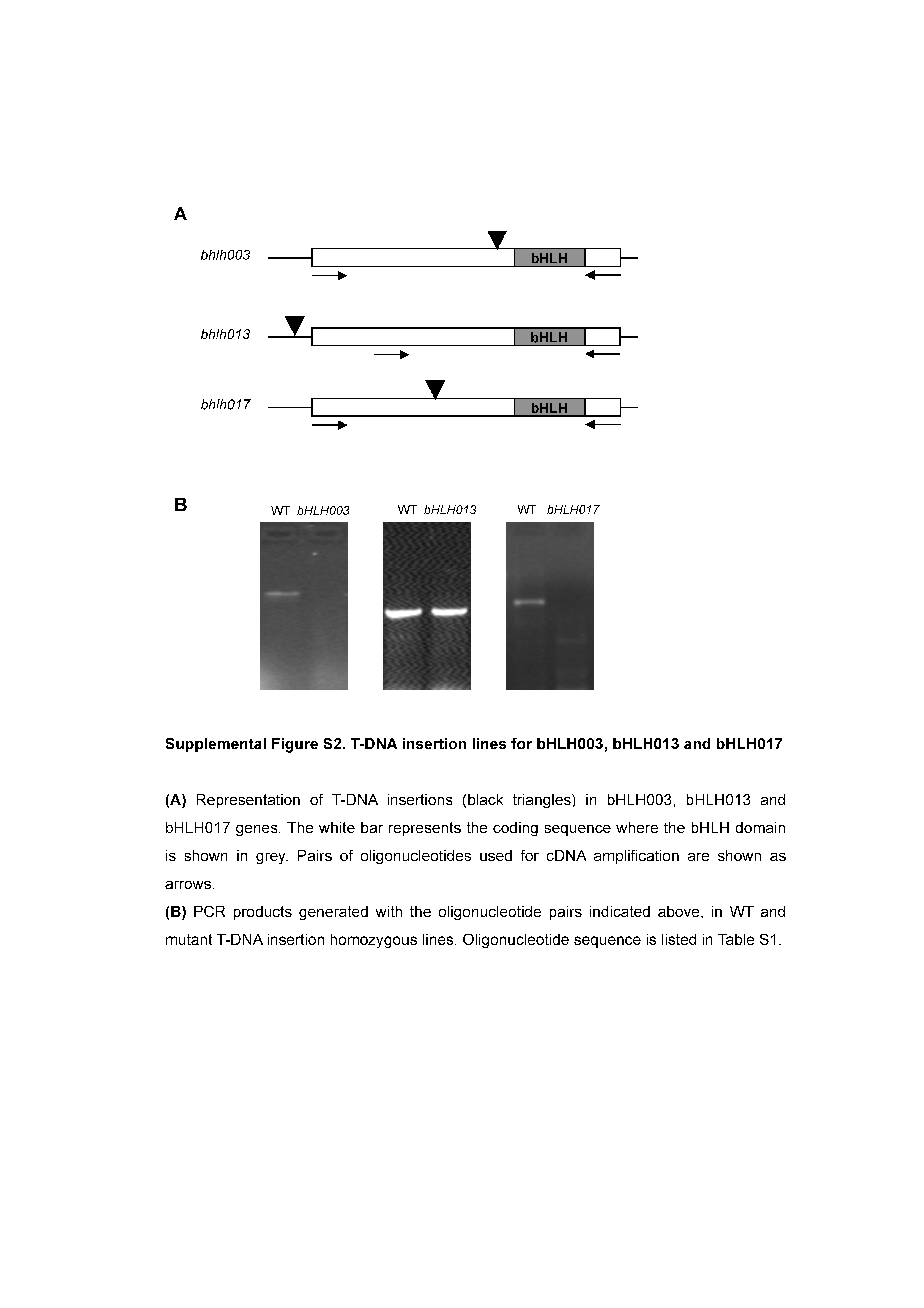

Supplement: Figure S2 — T-DNA insertion lines for bHLH003, bHLH013 and bHLH017. (A) Representation of T-DNA insertions (black triangles) in bHLH003, bHLH013 and bHLH017 genes. The white bar represents the coding sequence where the bHLH domain is shown in grey. Pairs of oligonucleotides used for cDNA amplification are shown as arrows. (B) PCR products generated with the oligonucleotide pairs indicated above, in WT and mutant T-DNA insertion homozygous lines. Oligonucleotide sequence is listed in Table S1. (TIFF) [file pone.0086182.s002.tiff]

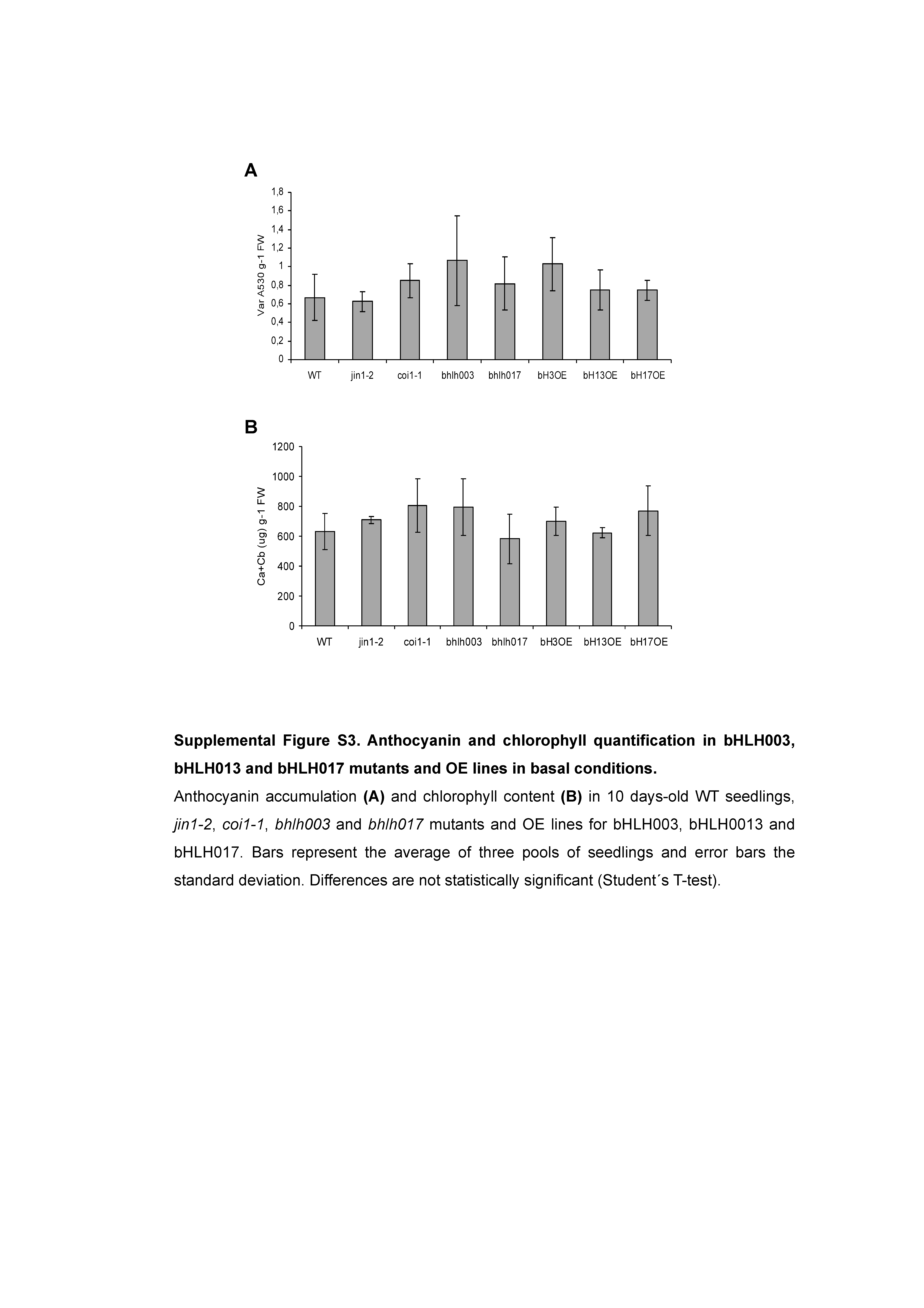

Supplement: Figure S3 — Anthocyanin and chlorophyll quantification in bHLH003, bHLH013 and bHLH017 mutants and OE lines in basal conditions. Anthocyanin accumulation (A) and chlorophyll content (B) in 10 days-old WT seedlings, jin1-2, coi1-1, bhlh003 and bhlh017 mutants and OE lines for bHLH003, bHLH0013 and bHLH017. Bars represent the average of three pools of seedlings and error bars the standard deviation. Differences are not statistically significant (Student's T-test). (TIFF) [file pone.0086182.s003.tiff]
